# Transcriptome analysis reveals chrysanthemum flower discoloration under high-temperature stress

**DOI:** 10.3389/fpls.2022.1003635

**Published:** 2022-09-14

**Authors:** Zhenjie Shi, Xiaoying Han, Guohui Wang, Jing Qiu, Li-jie Zhou, Sumei Chen, Weimin Fang, Fadi Chen, Jiafu Jiang

**Affiliations:** Sanya Institute of Nanjing Agricultural University, State Key Laboratory of Crop Genetics and Germplasm Enhancement, Key Laboratory of Landscaping, Ministry of Agriculture and Rural Affairs, Key Laboratory of Biology of Ornamental Plants in East China, National Forestry and Grassland Administration, College of Horticulture, Nanjing Agricultural University, Nanjing, China

**Keywords:** chrysanthemum, anthocyanin biosynthesis, high temperature, flower discoloration, RNA-Seq

## Abstract

Temperature is an important environmental factor affecting plant anthocyanin synthesis. High temperatures are associated with decreased anthocyanin pigmentation in chrysanthemum. To reveal the effects of high temperature on anthocyanin biosynthesis in chrysanthemum, ray florets of the heat-sensitive cultivar “Nannong Ziyunying” (ZYY) were subjected to RNA sequencing. A total of 18,286 unigenes were differentially expressed between the control and treatment groups. Functional annotation and enrichment analyses of these unigenes revealed that the heat shock response and flavonoid pathways were significantly enriched, suggesting that the expression of these genes in response to high temperature is associated with the fading of chrysanthemum flower color. In addition, genes related to anthocyanin synthesis and heat shock response were differentially expressed under high-temperature stress. Finally, to further investigate the molecular mechanism of discoloration under high-temperature stress and facilitate the use of marker-assisted breeding for developing novel heat-tolerant cultivars, these results were used to mine candidate genes by analyzing changes in their transcription levels in chrysanthemum.

## Introduction

With the intensification of global warming, high-temperature stress has become a major environmental factor limiting the normal growth and development of plants. Elevated temperatures affect a series of physiological, biochemical, and developmental processes in plants, such as photosynthesis and respiration, pollen activity, membrane stability, and enzyme activity, which in turn affect yield and quality (Atkin and Tjoelker, 2003; Nagar et al., 2015; Dusenge et al., 2019; Zhu et al., 2021). Temperature tolerance varies greatly among different plants. For instance, the rise in global temperatures has produced little effect on plants native to the tropics and subtropics; meanwhile, chrysanthemum is a cold-tolerant but heat-labile ornamental plant that is greatly affected by high temperatures (Gins et al., 2019). In general, high-temperature stress leads to delay in flowering, fading of flower color, and variation in flower type in chrysanthemums. Among these, flower color is one of the most important ornamental characteristics, and anthocyanins are the key component for flower color. Temperature-mediated regulation of flower color and the flavonoid pathway has been extensively studied (Nozaki and Fukai, 2008; Van Der Ploeg and Heuvelink, 2015). However, only a few studies have explored the regulation of chrysanthemum discoloration under high-temperature stress.

**Figure 1:**
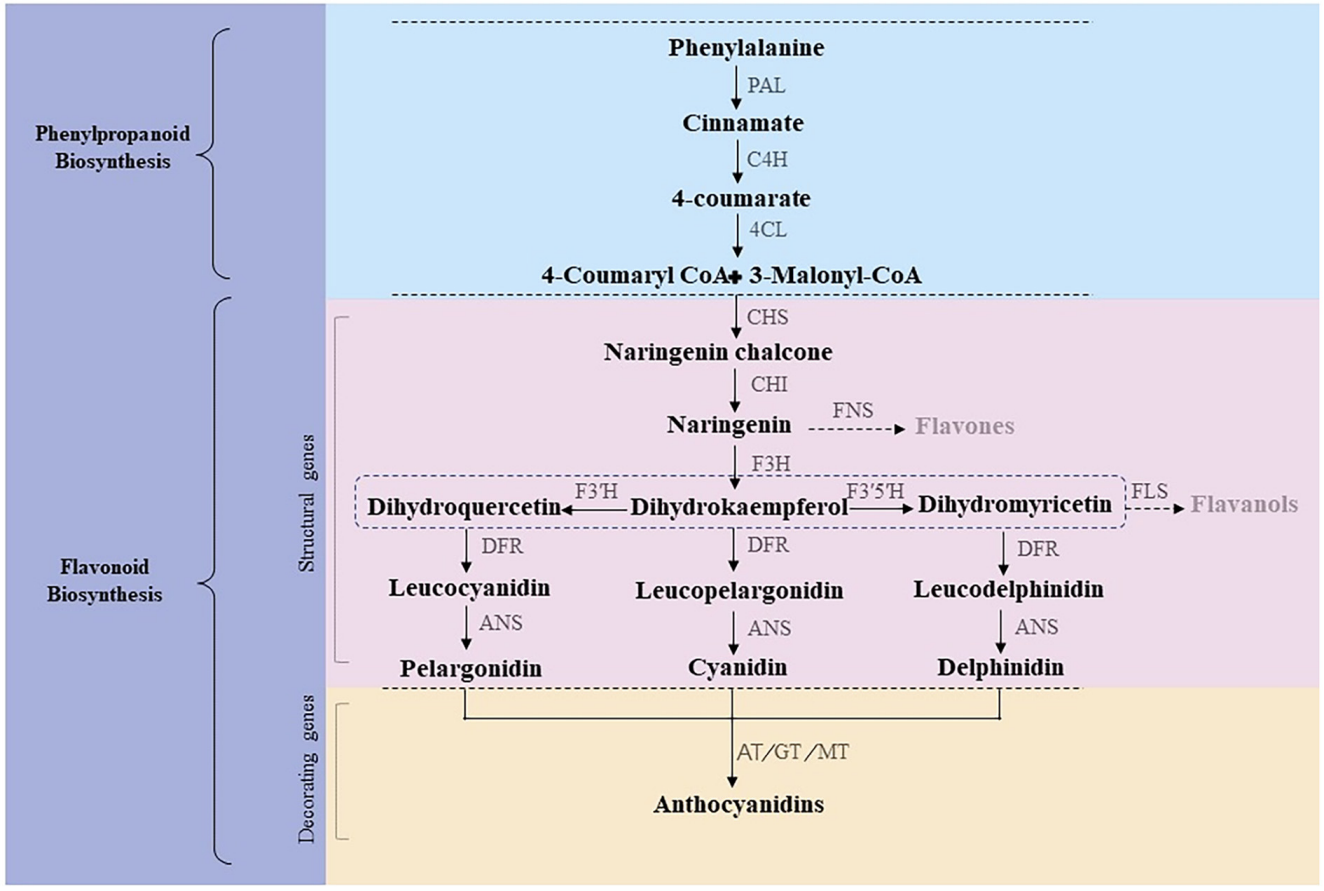
Anthocyanin synthetic pathway.

Anthocyanins are naturally synthesized water-soluble pigments present in various plant tissues and organs, such as roots, stems, leaves, flowers, fruits, and seeds. The anthocyanin synthetic pathway, or the phenylalanine pathway, is divided into three stages (Figure 1) (Winkel-Shirley, 2002). The first stage involves a phenylalanine precursor, which is oxidized by phenylalanine ammonia lyase (PAL), cinnamate 4-hydroxylase (C4H), and 4-coumaroyl CoA enzyme (4CL) to produce 4-coumaroyl-CoA. At the second stage, 4-coumaroyl-CoA is catalyzed by chalcone synthase (CHS) to produce chalcone, which is isomerized by chalcone isomerase (CHI) to form colorless trihydroxyflavonol. Finally, flavanone 3-hydroxylase (F3H), flavonoid-3',5'-hydroxylase (F3'5'H), and flavonoid-3'-hydroxylase (F3'H) catalyze the formation of colorless dihydroflavonols. At the third stage, colorless dihydroflavonols are further reduced to leucoanthocyanidins by dihydroflavonol 4-reductase (DFR) and then converted to anthocyanins by anthocyanin synthase (ANS) (Hichri et al., 2011; Zhang et al., 2016). Subsequently, glycosyltransferase (GT), methyltransferase (MT), and acyltransferase (AT) catalyze the formation of cornflower (cyanidin, Cy), geranium (pelargonidin, Pg), and delphinium (delphinidin, Dp) pigments (Caputi et al., 2012; Zhao et al., 2012; Bontpart et al., 2015). Of note, chrysanthemums lack the delphinium synthetic pathway; thus, there are no blue chrysanthemums in nature (Noda et al., 2017). Structural genes encoding *CHS, CHI, DFR, F3H, ANS*, and *3GT* involved in anthocyanin synthesis have been verified in several species (Shi et al., 2015; Sun et al., 2016; Li et al., 2020).

In recent years, several transcription factors involved in anthocyanin biosynthesis have been elucidated. For instance, MYB, bHLH, WD40, MADS-box and bZIP transcription factors are related to flower color. Structural genes involved in flavonoid pathways are primarily regulated by the conserved MYB–bHLH–WD40 (MBW) complex (Nabavi et al., 2020), which directly regulate flavonoid metabolism in plants through the transcriptional activation or repression of related structural genes. Among these, the transcription factor R2R3–MYB, which is the core component of the MBW protein complex, is mainly responsible for regulating flavonoid metabolism in plants (Xu et al., 2015). The MADS box proteins are a large family of transcription factors (TF) that control various developmental processes, not only the MADS box gene family has been involved in floral organogenesis according to the ABCDE model. However, MADS box TFs also regulate anthocyanin biosynthesis (Li et al., 2016; Qi et al., 2022). Further, bZIP transcription factors are involved in many signaling pathways, such as light signaling, environmental stress responses, and biological processes of plant growth and development (Chinnusamy et al., 2004; Golldack et al., 2014; An et al., 2019). HY5 is an important bZIP transcription involved in anthocyanin synthesis. It affects anthocyanin synthesis and metabolism by regulating the promoter activity of anthocyanin synthetic structural genes, including *CHS, CHI, F3H, F3'H, DFR*, and *ANS* (Zhang et al., 2011). Additionally, anthocyanin biosynthesis is affected by environmental factors, such as light and temperature. Temperature is crucial for anthocyanin metabolism. As such, the red color of maple leaves fades from the cooler northern regions to the warmer southern regions of the United States. This is because higher temperatures improve respiration, promote sugar consumption, and inhibit anthocyanin accumulation, leading to leaf color fading in plants (Deal et al., 1990). Furthermore, temperature affects the transcriptional expression of various genes in plants. Anthocyanin accumulation in marigold leaves is related to temperature, and accumulated temperatures are negatively correlated with marigold anthocyanin content (Armitage and Carlson, 1981). Under low temperatures, anthocyanin synthesis-related genes are induced and activated, elevating anthocyanin content in plants. In apple fruit, GT activity is significantly increased at low temperatures, increasing anthocyanin content beyond the normal value (Ubi et al., 2006). However, under high temperature, genes related to anthocyanin synthesis are inhibited, thereby reducing anthocyanin content (Islam et al., 2005). In plum fruits, over 60–70% anthocyanins are degraded under high temperature (Niu et al., 2017). Recently, a novel atypical subgroup 7 (SG7) R2R3–MYB transcription factor, CmMYB012, was discovered in chrysanthemum, which blocks anthocyanin biosynthesis by inhibiting *CmCHS, CmDFR, CmANS*, and *CmUFGT* expression under high-temperature stress (Zhou et al., 2021). Chrysanthemums can grow normally at 18–25°C; above 32°C, however, growth and development are delayed. In the temperature-sensitive chrysanthemum cultivars “Nannong Sichengdian,” “Nannong Ziyunying,” and “Nannong Zizhu,” flower color fades and ornamental quality declines under heat stress (Qiu, 2018). Abiotic stresses affect anthocyanin synthesis through several pathways. To date, the molecular mechanisms of chrysanthemum anthocyanin synthesis and flower discoloration in high-temperature environments remain unclear and warrant further exploration.

When plants are subjected to high-temperature stress, a series of heat shock response (HSR) pathways are activated, including the antioxidant protection system, thermoprotectant synthesis, membrane lipid peroxidation, heat shock protein expression, hormone regulation and signaling, and transcriptional regulation (Hasanuzzaman et al., 2013). Heat shock transcription factors (HSFs) and heat shock proteins (HSPs) play central roles in high-temperature stress response and acquired thermotolerance in plants (Zhang et al., 2015). HSFs act as key regulators of heat stress response. Specifically, they serve as transcriptional activators of HSPs, which are the terminal components of signal transduction pathways and mediate the expression of HSPs (Qu et al., 2013). HsfA1s are key transcriptional regulators in HSR (Ohama et al., 2017). Moreover, HSPs play pivotal roles in heat tolerance and are essential for the normal growth and development of plants under high temperatures. HSPs function as molecular chaperones and are classified into five families based on their molecular weight: HSP100, HSP90, HSP70, HSP60, and small HSPs (sHSPs) (Whitley et al., 1999; Tsan and Gao, 2004). Ca^2+^ and reactive oxygen species (ROS) are involved in signaling pathways that connect heat stress sensors and transcriptional regulators (Kotak et al., 2007). As one of the first signals in HSR, Ca^2+^ influx increases the strength of response (Liu et al., 2005). This alters plasma membrane (PM) fluidity, which in turn regulates cyclic nucleotide-gated calcium channels (CNGCs), allowing Ca^2+^ to enter the cytoplasm. CNGCs activate HSR by regulating the transition of cAMP and cGMP. As a Ca^2+^ signaling transmitter, CaM3 interacts with phosphorylated genes in response to heat stress (Finka et al., 2012; Tunc-Ozdemir et al., 2013). Additionally, nitric oxide (NO) has been reported to improve plant heat tolerance. In particular, NO acts as a signal upstream of CAM3 under heat stress (Rai et al., 2020). Furthermore, oxidative stress induced by HSR acts as secondary stress, which mediates ROS generation and causes molecular and cellular damage (Qu et al., 2013). The generated ROS are sensed by HSFs, which activate mitogen-activated protein kinase (MAPK) signaling, ultimately leading to the synthesis of antioxidant enzymes (SOD, POD, APX, CAT, and GPX) (Martindale and Holbrook, 2002). The MAPK cascade is an important signal transduction pathway in plants, being closely related to growth, development, and stress response (Kumar et al., 2020).

Here, using RNA sequencing (RNA-Seq), we found that anthocyanin synthesis-related genes, including *CHS, CHI, DFR, F3H, ANS, 3GT*, and *F3'H*, were significantly downregulated by heat in the temperature-sensitive cultivar “Nannong Ziyunying.” Moreover, MYB, bHLH, WD40, and bZIP transcription factors that regulate flavonoid metabolism and genes related to heat stress response-related processes, such as Ca^2+^ signaling, MAPK cascade, HSF and HSP function, and ROS generation, were differentially expressed between heat-treated and untreated plants. Overall, the present study aimed to explore molecular mechanisms underlying anthocyanin synthesis under high-temperature stress.

## Materials and methods

### Plant material and growth conditions

The cut-flower chrysanthemum cultivars “Nanong Ziyunying” and “Nannong Zizhu” were obtained from the China Chrysanthemum Germplasm Resources Preservation Center of Nanjing Agricultural University. Cuttings were rooted in trays for 15 days, transplanted into pots, grown for a month at a suitable temperature (day/night = 24°C/18°C), and cultured under a 16/8 h light/dark cycle and 70% relative humidity until the bud period. Thereafter, two groups of plants were transferred to separate light incubators with different environments: control group (CK) (8/16 h light/dark cycle with day and night temperature of 22°C) and high-temperature treatment group (8/16 h light/dark cycle with day/night temperatures of 38°C/22°C). Both groups of plants were watered every 5 days during the treatment period. The two cultivars with different treatments were sampled at the bud, early bloom, and bloom stages at the same period. Three biological and three technical replicates were collected for each sample and stored in liquid nitrogen for subsequent high-throughput sequencing assays.

### Determination of anthocyanin type and content

#### Determination of anthocyanin type

Qualitative analysis was performed to determine the anthocyanin type in high-temperature-treated cultivars. Briefly, the ray florets of “Nannong Ziyunying” and “Nannong Zizhu” were collected; each fresh sample weighed approximately 0.1 g. Then, 5 mL of petroleum ether, 5 mL of 10% hydrochloric acid, and 5 mL of 30% ammonia water were added to the samples. Subsequently, the samples were rapidly ground to a slurry, and the supernatant was collected after centrifugation (at 5,000 rpm for 10 min) for analysis. The type of anthocyanin component was determined based on color change.

#### Determination of anthocyanin content

Anthocyanin content of high-temperature-treated cultivars was determined as follows. At the flowering stage, ray florets of both cultivars from different treatment groups were collected. Next, 0.2 g of each sample was weighed and cryogenically ground to a powder. Then, 5 mL of solvent (methanol:water:formic acid:trifluoroacetic acid = 70:27:2:1) was added to each sample, and the extracts were shaken thoroughly. After 24 h, the samples were centrifuged (at 5,000 rpm for 10 min), and the supernatant was collected for analysis. Absorbance was measured at 530 and 657 nm using a spectrophotometer (Puana/T6 New Century). Each sample was analyzed three times.

Anthocyanin content was calculated according to the following formula:


$$\begin{array}{l} \text{Anthocyanin content}=\text{OD}/\text{g}\cdot \text{FW}\\ \Delta \text{OD}=\text{O}{\text{D}}_{530}-1/4\text{O}{\text{D}}_{657} \end{array}$$

### Total RNA extraction, library construction, and RNA-Seq

Total RNA was extracted using RNA isoPlus (TaKaRa, Japan). The quality of extracted RNA was determined using agarose gel electrophoresis, and RNA quality and concentration were confirmed using a spectrophotometer (ND-1,000; NanoDrop, Wilmington, DE). High-throughput sequencing was performed using the BGISEQ-500 platform.

The basic steps and data processing methods for transcriptome sequencing were as follows. First, total RNA was processed for mRNA enrichment and rRNA depletion. DNA/RNA hybrid strands were selectively digested using RNase H, the remaining DNA probe was digested with DNase I, and desired RNA was obtained after purification. Next, RNA was fragmented in a specific buffer and then reverse transcribed with N6 random primers to synthesize double-stranded DNA; ends of the DNA fragments were blunted, modified, and PCR-amplified with specific primers. The obtained PCR products were thermally denatured into single strands and circularized with bridge primers to obtain a single-stranded circular DNA library for sequencing. Finally, high-quality clean reads were obtained after filtering the raw reads. Clean reads were assembled using Trinity and TGICL to remove redundancy and splicing, and the final unigenes were obtained by matching with the reference genome. Functional annotation and simple sequence repeat (SSR) detection of unigenes, simultaneous calculation of differentially expressed genes (DEGs) for each sample, and in-depth cluster and functional enrichment analyses were performed as described below.

### DEG analysis, gene function annotation, GO and KEGG enrichment

BLAST (https://blast.ncbi.nlm.nih.gov/Blast.cgi) was used to search the assembled unigenes against the NCBI NR (non-redundant protein sequences), Pfam (protein families), Swiss-Prot (a manually annotated and reviewed protein sequences), Kyoto Encyclopedia of Genes and Genomes (KEGG), Cluster of Orthologous Groups (COG), NCBI NT (nucleotide sequences), Gene Ontology (GO), and Eukaryotic Orthologous Groups (KOG) functional databases. The GO functions of all unigenes were classified using WEGO. According to the KEGG annotation information, metabolic pathways of the unigenes were explored. DEGs were detected using the PossionDis method. The Q-value was used to correct for the significant *p*-values obtained in original hypothesis tests. To minimize the false-positive rate, a Q-value<0.01 and |log_2_ fold change|>1 were set as the thresholds for filtering DEGs. A log_2_ fold change>0 indicated an upregulated gene; otherwise, it was considered downregulated.

### Quantitative real-time PCR

Light Cycler 480 was used for qRT-PCR on DEGs to verify the reliability of the transcriptomic data. The specific primers used for qRT-PCR were designed using Primer Premier 5 (Table 1). Three technical and three biological replicates were set for each test sample. The reactions were performed using SYBR Premix Ex Taq^TM^ (TaKaRa, Japan) according to the manufacturer's instructions. *CmEF1*α was selected as the reference gene. Relative gene expression was assessed using the 2^−ΔΔCT^ method.

**Table 1:** Primers used in the study.

**Primer name**	**Sequence (5'-3')**
qCmCHS-F	ATCATCCAATGATGGTGCCATATAGGC
qCmCHS-R	GACCGCTACACCTCCTAATTGTGTACT
qCmCHI-F	ATACCTGAAGCACCAATCGCAGT
qCmCHI-R	ATTTTCCTCTAGTTGAAAAAGCC
qCmF3H-F	CCAACATTCATCATTCATCTCCCCAGA
qCmF3H-R	ACTTGACAACAACCACTTTCAGGGAGG
qCmDFR-F	CGGAGAAAGCAGCATGGAAA
qCmDFR-R	GGGAACGAGGGACTGATAAATG
qCmANS-F	ATCAACTACTACCCAAAATGCCC
qCmANS-R	CCTAACCTTCTCCTTATTCACAA
qCm3GT-F	GGAGAAATGGAAGATAAACCGAA
qCm3GT-R	CGCCGAATAAAGGAAATCCTAAG
qCmEF1α-F	GTACCCTGGGCACCACAAGT
qCmEF1α-R	CTACCAACGGCCTGCAAATC
qCmHY5-F	GGCTGACAAAGAAAACAAACGGT
qCmHY5-R	CGTTTTGCAGTGTGGACAAACGC

## Results

### Phenotypic analysis of “Nannong Ziyunying” and “Nannong Zizhu” under high-temperature stress

**Figure 2:**
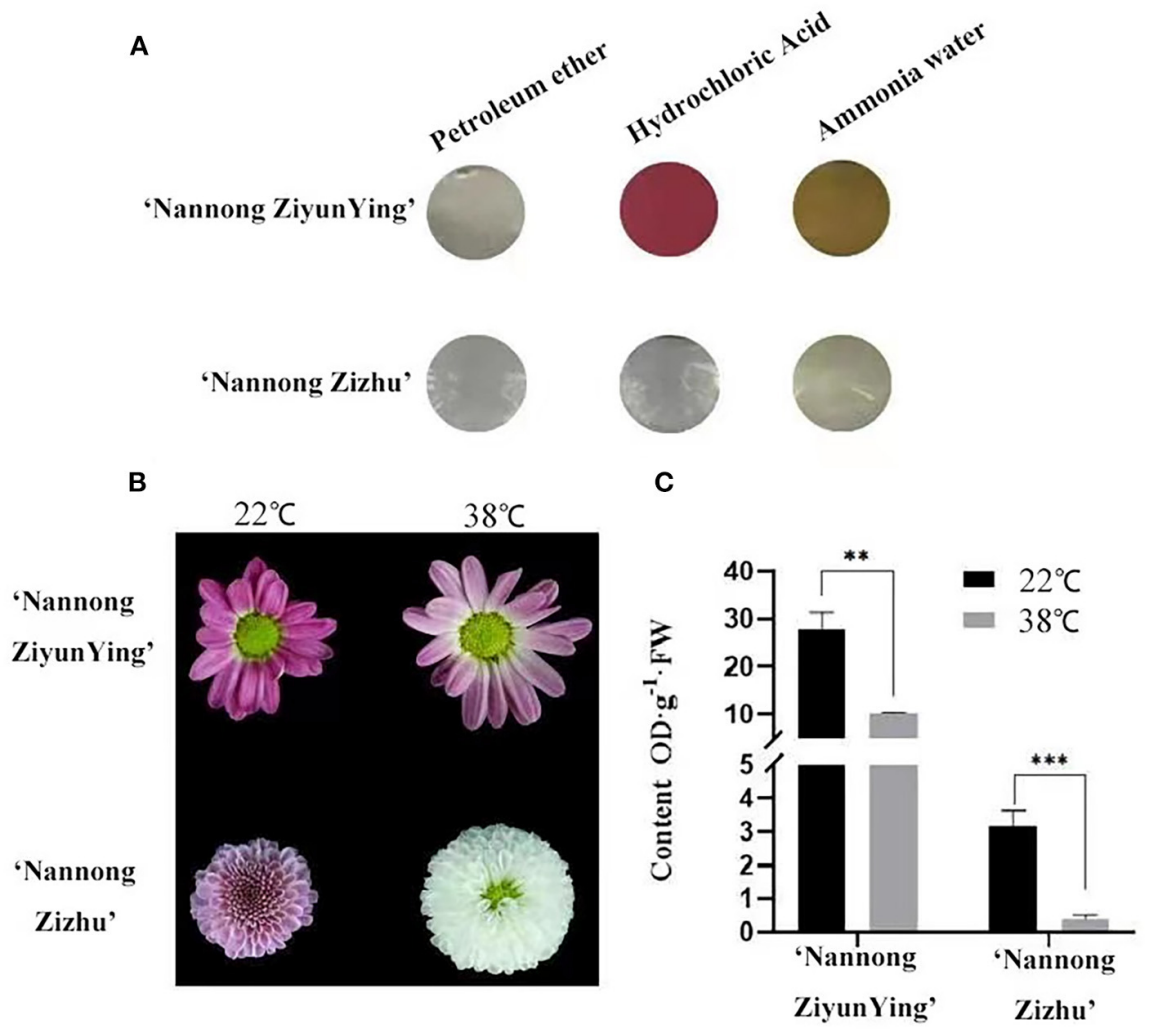
Qualitative analysis of anthocyanin types and content in two cut chrysanthemum cultivars. **(A)** Calibration of anthocyanin components. **(B)** Comparison of high-temperature-treated and untreated flower color phenotypes. **(C)** Anthocyanin content of high-temperature treated and untreated cultivars. Error bars represent standard deviations from three biological replicates. Values are presented as mean ± SE (*n* = 3). **Significant at *P* < 0.01 and ***extremely significant at *P* < 0.001, as determined by Student's *t*-test.

The chrysanthemum cultivars “Nannong Ziyunying” and “Nannong Zizhu” were easily affected, and their flower color changed under high-temperature stress. We analyzed the anthocyanin types of the two cultivars and found that the pigments were anthocyanins and flavonoids but not carotenoids (Figure 2A). Thus, we hypothesized that temperature affects anthocyanin synthesis during flowering in “Nannong Ziyunying” and “Nannong Zizhu” subjected to heat stress. In addition, the flower color of both cultivars faded following high-temperature treatment (Figure 2B). To explore the correlation between flower color and anthocyanin content, we measured anthocyanin content in treated and untreated fresh ray florets. The total anthocyanin content in the control and treatment groups was, respectively 27.89 and 10.19 OD·g^−1^·FW for “Nanong Ziyunying” and respectively, 3.16 and 0.38 OD·g^−1^·FW for “Nannong Zizhu.” Thus, in both cultivars, the total anthocyanin content of ray florets was significantly reduced after high-temperature treatment (Figure 2C).

### Transcriptome analysis after high-temperature treatment

**Figure 3:**
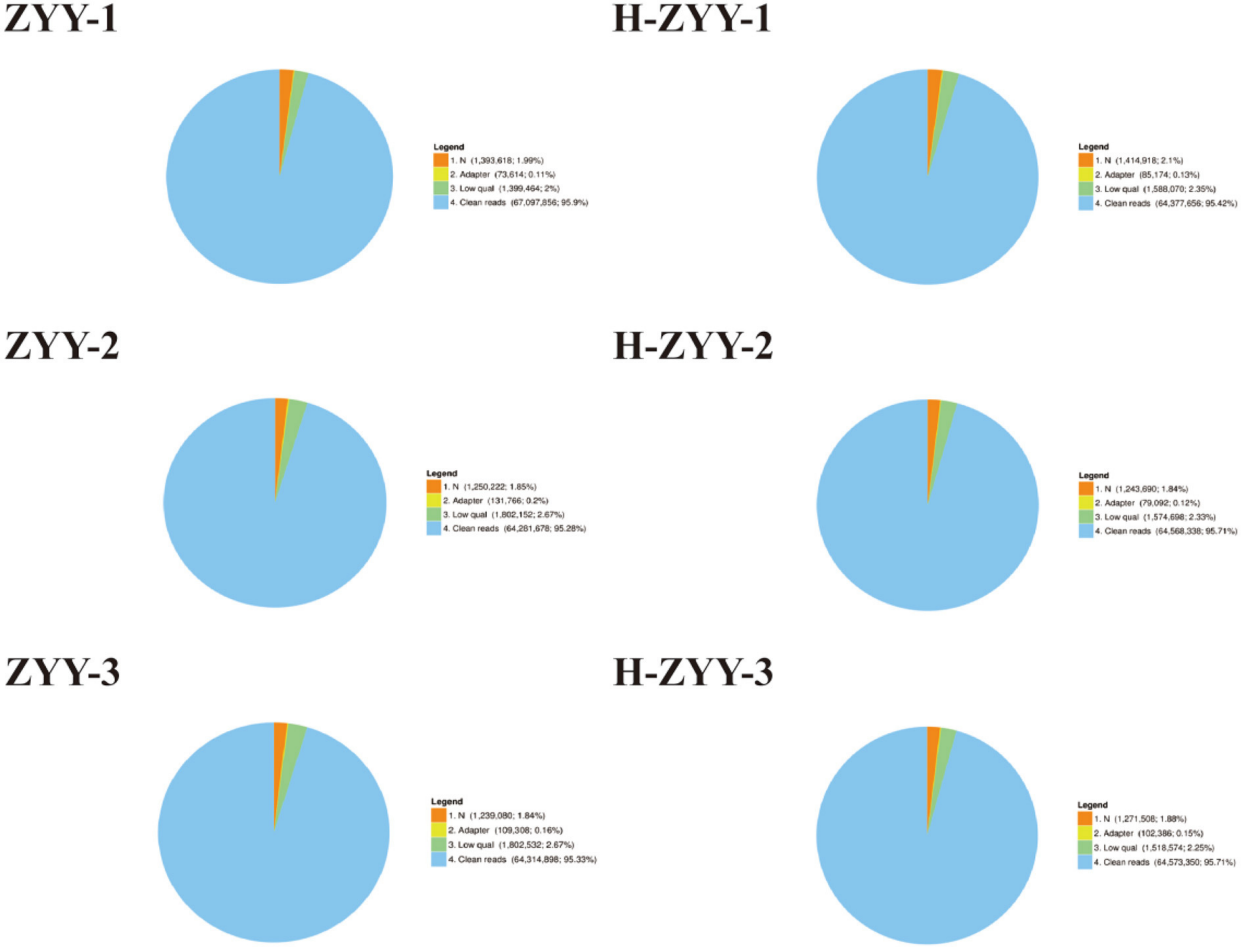
Comparison of raw reads in six RNA libraries. “Clean reads” represent the elimination of reads containing adaptor; “N” represents reads accounting for over 10%; “low quality” represents remaining reads after the number of bases with quality value (Q) ≤ 5 is over 50% of all reads. Numbers in brackets indicate the percentage of reads of each type.

The heat-sensitive cultivar “Nanong Ziyunying” was selected as the material for transcriptome analysis. Fresh ray florets were subjected to high-temperature and normal conditions, and high-quality RNA was extracted. Three biological replicates were used per treatment group. The control groups were designated ZYY-1, ZYY-2, and ZYY-3, and the high-temperature treatment groups were designated H-ZYY-1, H-ZYY-2, and H-ZYY-3. After sequencing, raw data were filtered and processed for statistical analysis (Table 2). Overall, the six databases contained a low proportion of low-quality bases (quality<20) and the sequencing quality was good (Figure 3).

**Table 2:** Filtered read quality statistics.

**Sample**	**Total raw reads (M)**	**Total clean reads (M)**	**Total clean bases (Gb)**	**Clean reads Q20 (%)**	**Clean reads Q30 (%)**	**Clean read ratio (%)**
ZYY-1	69.96	67.1	6.71	98.22	92.16	95.9
ZYY-2	67.47	64.28	6.43	98.18	92.1	95.28
ZYY-3	67.47	64.31	6.43	98.15	91.94	95.33
H-ZYY-1	67.47	64.38	6.44	98.22	92.2	95.42
H-ZYY-2	67.47	64.57	6.46	98.17	92.02	95.71
H-ZYY-3	67.47	64.57	6.46	98.19	92.06	95.71

A total data of 38.93 GB of data were generated from the BGISEQ-500 platform. After assembly and de-redundancy, 151,614 unigenes were obtained. The total length, average length, N50, and GC content were 154,407,019 bp, 1,018 bp, 1,561, and 39.36%, respectively (Table 3). The obtained unigenes were annotated against the seven functional databases using BLAST, and respectively 93,977 (NR: 61.98%), 56,053 (NT: 36.97%), 63,543 (Swiss-Prot: 41.91%), 72,078 (KOG: 47.54%), 69,096 (KEGG: 45.57%), 73,203 (GO: 48.28%), and 63,728 (Pfam: 42.03%) unigenes were functionally annotated. The transdecoder detected 72,964 coding sequences and 20,880 SSRs distributed in 17,391 unigenes and predicted 2,742 unigenes encoding transcription factors.

**Table 3:** Unigene quality index statistics.

**Sample**	**Total number**	**Total length**	**Mean length**	**N50**	**N70**	**N90**	**GC (%)**
ZYY-1	98,773	77,777,880	787	1,176	718	337	39.58
ZYY-2	92,109	72,159,678	783	1,170	716	336	39.7
ZYY-3	97,167	75,886,636	780	1,163	712	334	39.59
H-ZYY-1	82,749	70,451,968	851	1,257	799	376	39.84
H-ZYY-2	82,070	69787,861	850	1264	794	374	39.9
H-ZYY-3	83,300	70,866,818	850	1,263	793	375	39.82
All-unigene	151,614	154,407,019	1,018	1,561	1,012	463	39.36

### Analysis of DEGs and functional enrichment after high temperature treatment

**Figure 4:**
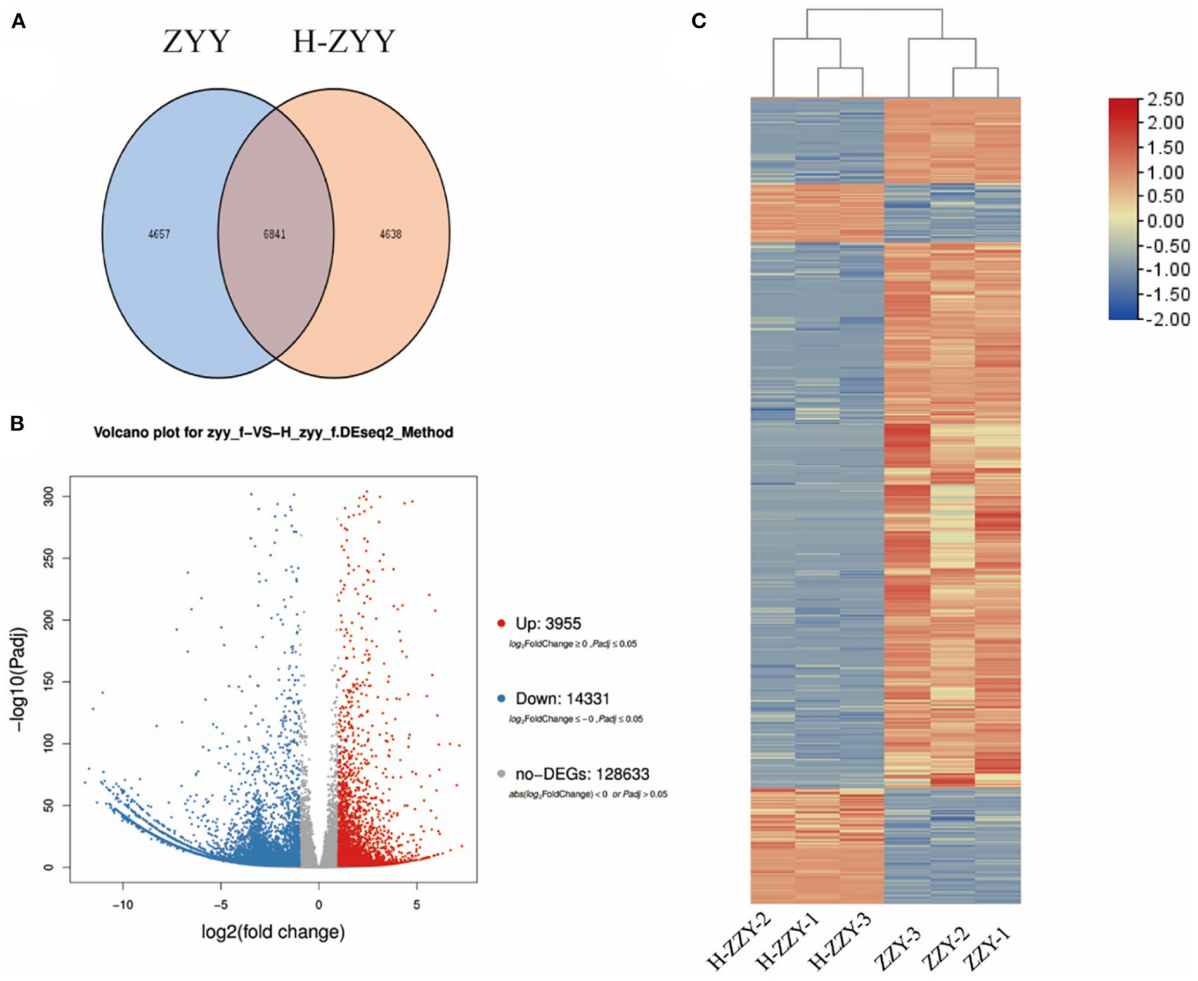
Analysis of differentially expressed genes. **(A)** Venn diagram. **(B)** Volcano plot. **(C)** Heatmap.

As shown in Figure 4, Venn diagrams of transcripts illustrated 6,841 co-expressed genes, 4,657 ZYY-specific, and 4638 H-ZYY-specific genes between the control (ZYY) and treated (H-ZYY) groups (Figure 4A). Analysis of transcriptome data revealed 18,286 DEGs between ZYY and H-ZYY, of which 14,331 unigenes were downregulated and 3,955 were upregulated in the treatment group (Figure 4B). Additionally, hierarchical clustering of DEGs was observed between ZYY (ZYY-1, ZYY-2, and ZYY-3) and H-ZYY (H-ZYY-1, H-ZYY-2, and H-ZYY-3) (Figure 4C).

**Figure 5:**
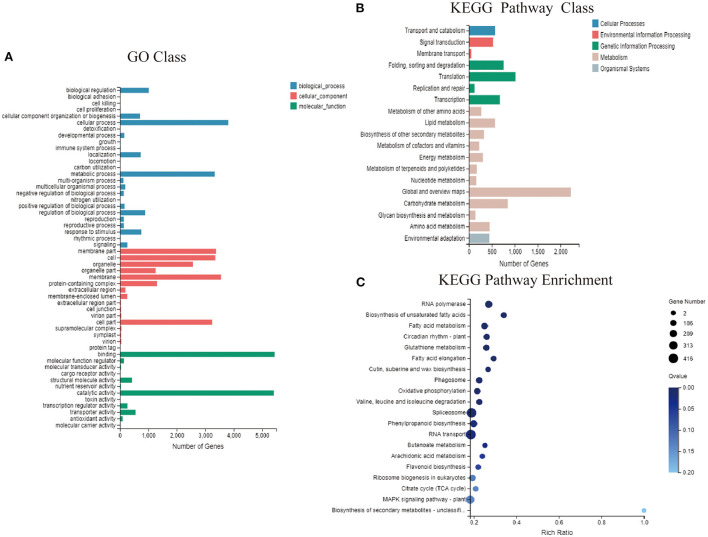
GO and KEGG functional enrichment analysis. **(A)** Functional distribution of GO annotations. **(B)** KEGG function distribution statistics. **(C)** KEGG function-enriched bubble chart.

The functional classification of GO annotations mainly included three categories, namely “molecular function” “cellular component” and “biological processes” corresponding to respectively, 15, 15, and 9 specific functions (Figure 5A). Among these, respectively 5,447 and 5,415 unigenes annotated to “molecular function” mainly focused on “catalytic activity” and “binding.” Moreover, 3,341 unigenes annotated to “biological processes” mainly focused on “cellular and metabolic processes.” Among unigenes annotated to “cell components,” 3,355, 3,241, 3,550, and 3,374 focused on “cell” “cell part” “membrane” and “membrane part,” respectively. Gene-related KEGG pathways were divided into five branches: “cellular processes” “environmental information processing” “genetic information processing” “metabolism” and “organismal systems” (Figure 5B). Most of the unigenes were functionally annotated to “metabolic pathways” which were divided into 11 sub-categories, with 2,224 genes in the global and overview maps and 847 genes in the carbohydrate metabolism category. In addition, unigenes classified into “genetic information processing” pathways were divided into four sub-categories focusing on “translation” and “folding, sorting, and degradation,” with respectively, 1,015 and 757 genes. Functional enrichment analysis revealed abundant unigenes in “RNA transport” “spliceosome” and “plant MPAK signaling” pathways. Of the 416, 408, and 306 DEGs, 63 were enriched in “flavonoid biosynthesis” which is associated with flower color (Figure 5C).

### RNA-Seq database mining of DEGs in response to high-temperature treatment

**Figure 6:**
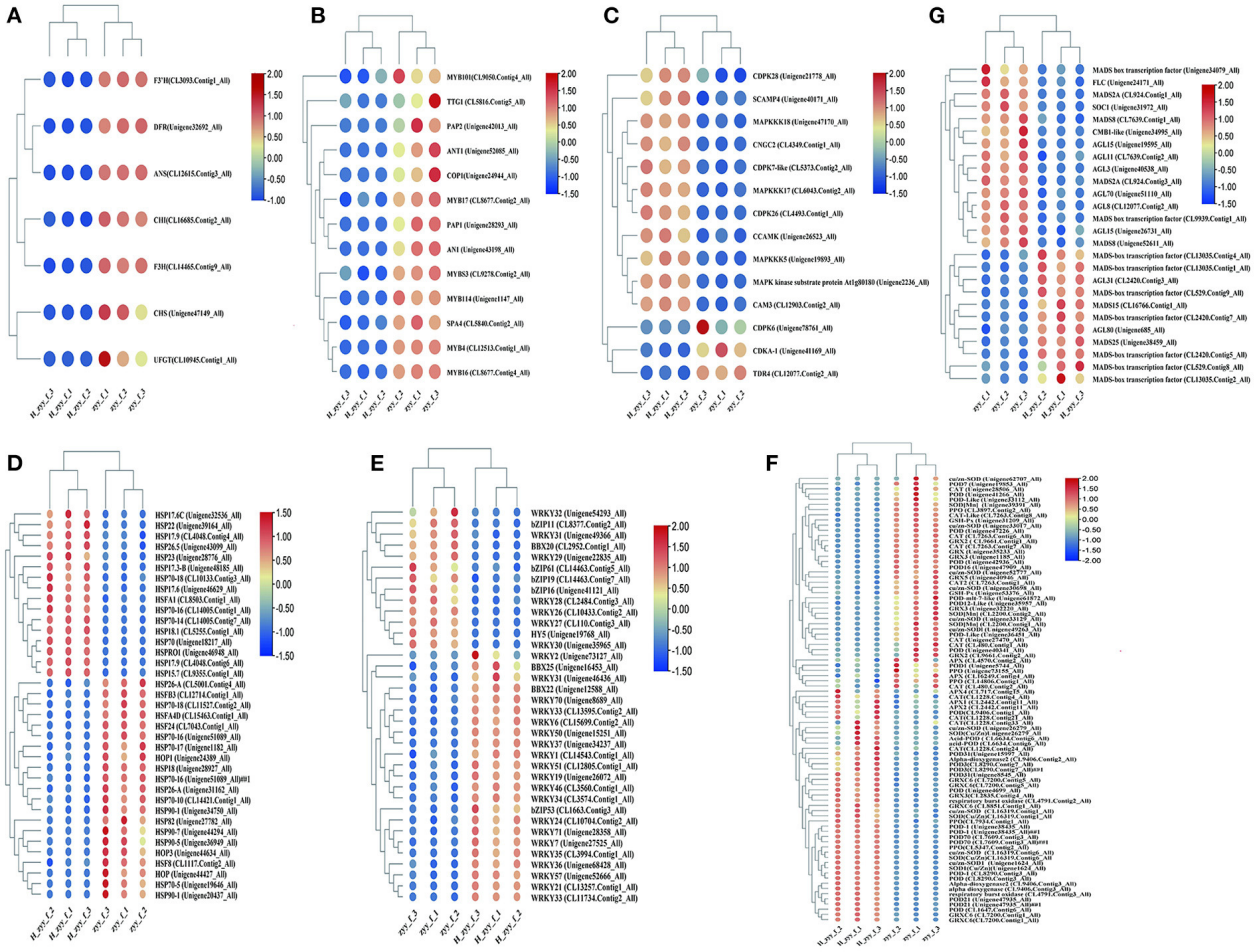
Mining of major pathways in response to high-temperature stress. **(A)** Anthocyanin metabolic pathway. **(B)** MBW transcription factors. **(C)** Ca^2+^ channels and Ca^2+^ signaling. **(D)** HSFs and HSPs. **(E)** Stress-responsive transcription factors. **(F)** ROS response pathway. **(G)** MADS-box transcription factors.

DEGs between the ZYY and H-ZYY groups were compared with the gene function annotation information, and the transcription factors and genes potentially involved in response to high-temperature treatment were extracted from the transcriptome database. Since the flower color of “Nannong Ziyunying” changed after high-temperature treatment, we performed differential analysis of genes related to flower color metabolism. The structural genes *CHS* (Unigene47149_All), *CHI* (CL16685.Contig2_All), *DFR* (Unigene32692_All), *F3H* (CL14465.Contig9_All), *ANS* (CL12615.Contig3_All), *3GT* (CL10945.Contig1_All), and *F3'H* (CL3093Contig1_All) were downregulated (Figure 6A). The MBW transcription factors, which are associated with flower color, were analyzed. Of these, nine MYB transcription factors; the bHLH transcription factor *AN1* (Unigene43198_All); and three WD40 transcription factors, namely *TTG1* (CL5816.Contig5_All), *COP1* (Unigene24944_All), and *SPA4* (CL5840.Contig2_All), were downregulated (Figure 6B). The expression levels of MADS-box transcription factors also changed in the two phenotypes of ray florets after high-temperature treatment. Among them, the expression of *AGL31* (CL2420.Contig3_All), *MADS15* (CL16766.Contig1_All), and *MADS25* (Unigene38459_All) etc., were upregulated, while *CMB1-like* (Unigene34995_All), *AGL15* (Unigene19595_All), *AGL3* (Unigene40538_All), *SOC1* (Unigene31972_All), *FLC* (Unigene24171_All), *AGL15* (Unigene26731_All), and *MADS8* (Unigene52611_All), were significantly downregulated (Figure 6G). Furthermore, signaling molecules, such as Ca^2+^ and ROS, accumulate in plants in response to high-temperature stress. Ca^2+^ is one of the primary and essential signals for heat shock response, and it activates MAPK to transmit signals to the nucleus (Ohama et al., 2017). Our analysis revealed that *MAPKKK17* (CL6043.Contig2_All), *MAPKKK5* (Unigene19893_All), the MAPK kinase substrate protein At1g80180 (Unigene2236_All), and *MAPKKK18* (Unigene47170_All) in the MAPK cascade were upregulated following high-temperature treatment. Likewise, the intracellular Ca^2+^ signaling pathway genes *CAM3* (CL12903.Contig2_All), *CCAMK* (Unigene26523_All), *SCAMP4* (Unigene40171_All), and *CNGC2* (CL4349.Contig1_Al) were upregulated, whereas *CDKA*-1 (Unigene41169_All) and *TDR4* (CL12077.Contig2_All) were downregulated (Figure 6C) after high-temperature treatment. Stress induces ROS production via both enzymatic and non-enzymatic pathways and is associated with multiple stages of plant development and growth. Our analysis revealed that several genes encoding antioxidant enzymes, such as *SOD[Mn]* (CL2200.Contig2_All), *Cu/Zn-SOD* (Unigene49263_Al), POD (Unigene40341_All), *POD-like* (Unigene36451_All), *POD16* (Unigene47909_All), *POD7* (Unigene19853_All), *CAT* (Unigene27470_All), *CAT2* (CL7263.Contig1_All), *APX* (CL16249.Contig4_All), *PPO* (CL3897.Contig2_All), *GRX2* (CL9661.Contig1_All), and *GRX3* (Unigene32220_All) were downregulated under high-temperature stress. However, *Cu/zn-SOD* (CL16319.Contig1_All), *POD31* (Unigene8545_All), *POD3* (CL8290.Contig7_All), *POD-1* (Unigene38435_All), *PPO* (CL7934.Contig1_All), CAT (CL1228.Contig24_All), *GRXC6* (CL7200.Contig1_All), *APX2* (CL2442.Contig11_All) were upregulated, indicating heat enhanced ROS metabolism (Figure 6F).

HSFs and HSPs are involved in various biological processes, particularly in response to heat stress. Specifically, the heat shock transcription factor (Hsf) *HsfA1* (CL8503.Contig1_All) was upregulated, while *HSF8* (CL1117.Contig2_All), *HSFB3* (CL12714.Contig1_All), *HSFA4D* (CL15463.Contig1_All), and *HSF24* (CL7043.Contig1_All) were downregulated. In addition, 16 HSPs were upregulated and 16 were downregulated after high-temperature treatment. Among these, *HSP70* (Unigene18217_All) was significantly upregulated, whereas *HSP90* (Unigene31147_All) was significantly downregulated (Figure 6D). In addition to HSFs and HSPs, transcription factors, such as B-box (BBX), bZIP, DREB, and WRKY, were differentially expressed. In particular, the BBX transcription factors *BBX22* (Unigene12588_All) and *BBX25* (Unigene16453_All) were upregulated, while *BBX20* (CL2952.Contig1_All) was downregulated. Among bZIP transcription factors, *bZIP53* (CL1663.Contig3_All), *bZIP61* (CL14463.Contig5_All), *bZIP19* (CL14463.Contig7_All), *bZIP11* (CL8377.Contig2_All), and *bZIP16* (Unigene41121_All) were upregulated, while *HY5* (Unigene19768_All) was significantly downregulated. DREB is an important stress-responsive transcription factor in plants, which can specifically bind *cis*-acting DRE/CRT elements to induce the expression of stress tolerance genes (Luo et al., 2015). Our analysis revealed that *DREB3* (CL11734.Contig2_All), *DREB1F* (CL3574.Contig1_All), *DREB1A* (CL3994.Contig1_All), *DREB1B* (Unigene68428_All), and *DREB1D* (Unigene34237_All) were upregulated in response to high temperature. In addition, 22 WRKY transcription factors were differentially expressed, of which 15 were upregulated and 7 were downregulated. Specifically, *WRKY2* (Unigene73127_All) was significantly upregulated, while *WRKY3* (CL110.Contig3_All) was significantly downregulated (Figure 6E).

### qRT-PCR validation of flower color-related DEGs

**Figure 7:**
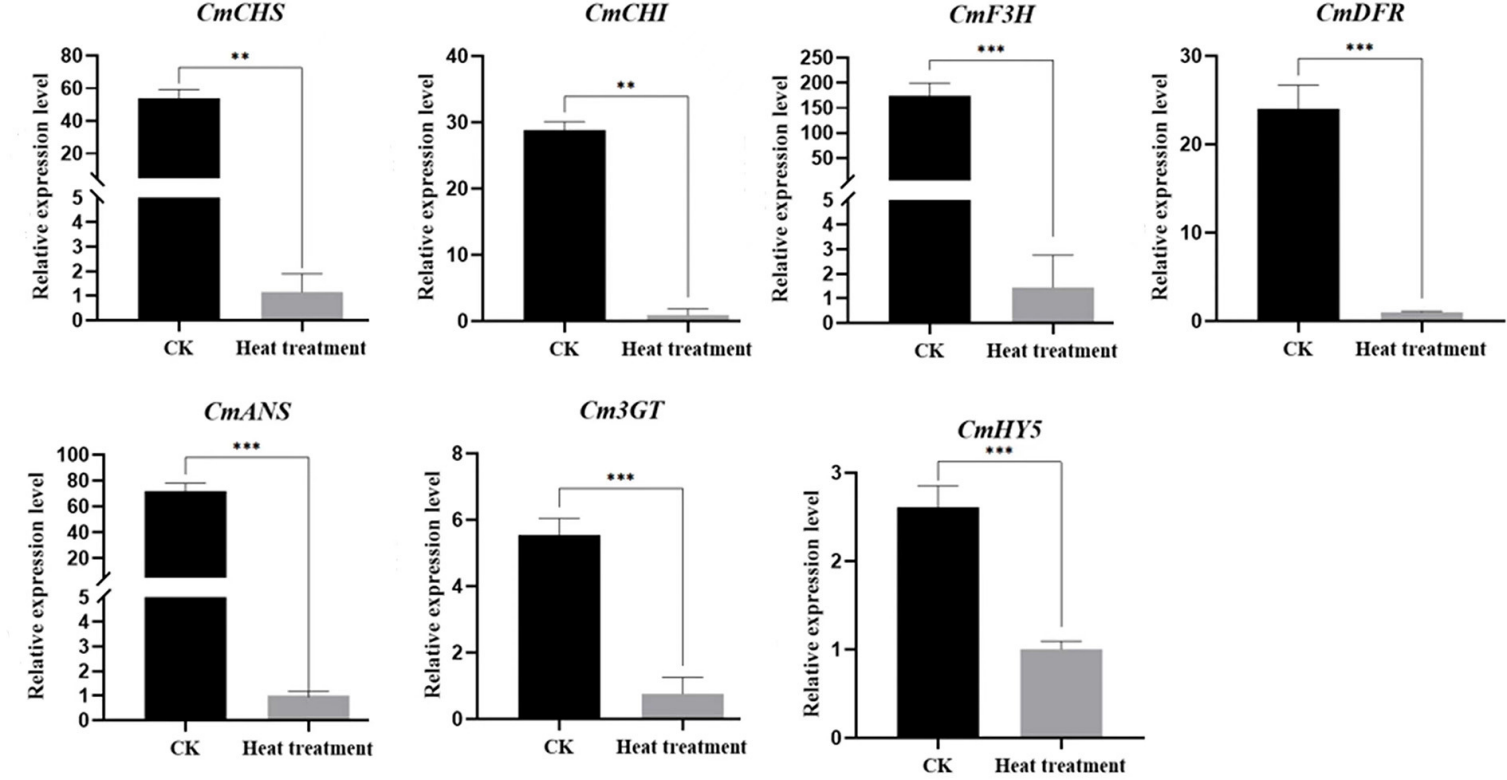
Verification of the relative expression of anthocyanin metabolism gene following high-temperature treatment. Values are presented as mean ± SE (*n* = 3). **Significant at *P* < 0.01 and ***significant at *P* < 0.001, as determined by Student's *t*-test.

To validate the expression of differential genes in the RNA-Seq database, we selected seven DEGs, namely *CmCHS, CmCHI, CmDFR, CmF3H, CmANS, Cm3GT*, and *CmHY5*, which are structural anthocyanin synthetic genes and transcription factors, for qRT-PCR analysis. The results of qRT-PCR were consistent with those of RNA-Seq, and all seven genes were significantly downregulated after high-temperature treatment (Figure 7).

## Discussion

### How does chrysanthemum respond to high-temperature stress?

Through transcriptome analysis, changes in flower color following high-temperature treatment of the heat-sensitive cultivar “Nannong Ziyunying” at the molecular level were revealed. Our analysis revealed that *HsfA1* (CL8503.Contig1_All) was significantly upregulated after high-temperature treatment. HsfA1 is a key regulator of response to high-temperature stress (El-Shershaby et al., 2019). For instance, in tomato, HsfA1 serves a unique function of regulating plant thermotolerance as a major transcription factor in response to heat stress, and *hsfa1* mutants exhibit a heat-sensitive phenotype (Mishra et al., 2002). In addition, *HSP70* (Unigene18217_All) expression was significantly upregulated after high-temperature treatment. HSP70 is the main and highly conserved protein activated by stress (Usman et al., 2017), and the role of HSP70 in relation to heat tolerance has been reported in several crops, such as soybean (*Glycine max*) (Ortiz and Cardemil, 2001), wheat (*Triticum aestivum*) (Duan et al., 2011), chili pepper (*Capsicum annuum*) (Usman et al., 2015), chrysanthemum (*Chrysanthemum morifolium* Ramat) (Song et al., 2014), and creeping bentgrass (*Agrostis palustris*) (Ye et al., 2012). The same results were obtained in our experiments, indicating that HSFs and HSPs respond to high-temperature stress in chrysanthemum.

In addition, high-temperature stress promotes the fluidity of cell membrane, which is related to Ca^2+^ channels in the cell membrane structure. Genes involved in Ca^2+^ signaling are activated under high-temperature stress, and heat shock signals are transmitted from the outside to inside of cells through CNGCs, which are implicated in the regulation of Ca^2+^ signaling (Finka et al., 2012). In particular, CNGC2 and CNGC4 are associated with high-temperature stress response (Gao et al., 2012). Our analysis revealed that *CNGC2* (CL4349.Contig1_All) was upregulated after high-temperature treatment, suggesting that Ca^2+^ signaling is activated under heat stress in chrysanthemum. In the Ca^2+^ signaling pathway, Ca^2+^ ions combine with calmodulin (CaM) to promote the activity of calcium-dependent protein kinases (CDPKs) and MAPKs, which transmit heat stress signals to the nucleus and regulate the expression of thermostable genes (Awasthi et al., 2015). According to our transcriptomic data, MAPKs (Unigene2236_All) and CAM3 (CL12903.Contig2_All) were upregulated after high-temperature treatment. In addition, high-temperature stress induces ROS production and accumulation in plants, promoting antioxidant enzyme activity. In the present study, the expression levels of *SOD, POD, CAT, GRX, PPO*, and *APX* were significantly altered (Figure 6F). Increased activity of these antioxidant enzymes is beneficial for scavenging different types of ROS [e.g., singlet oxygen (^1^O_2_), superoxide (${\text{O}}_{2}^{-}$), hydrogen peroxide (H_2_O_2_), and hydroxyl radicals (OH^−^)] generated by high-temperature stress and enhancing the heat tolerance of plants (Medina et al., 2021). In addition, polyphenol oxidase (PPO) and peroxidase (POD) have been implicated in the anthocyanin degradation pathway for regulating flower and fruit color (Oren-Shamir, 2009; Zhang et al., 2019). ROS metabolism, similar to Ca^2+^ signaling pathway, mediates heat response and regulates immune activity in plants. Furthermore, ROS promote the expression of heat shock transcription factors; however, excess ROS accumulation leads to the generation of large amounts of NO, which induces the expression of CaM3 and promotes the transcription of downstream HsfA1 and related HSP genes (Ohama et al., 2017; Zhu et al., 2021). Furthermore, NO possesses ROS scavenging activity, and it has been reported to improve the antioxidant and thermal tolerance of *Arabidopsis* (Wang et al., 2014) and chrysanthemum (Yang et al., 2011). Typically, when plants are subjected to high-temperature stress, a series of transcriptional regulatory cascades are activated to respond to heat shock. HSF and HSP expression, Ca^2+^ signaling activation, and ROS production are the three major regulatory networks involved in HSR (Ohama et al., 2017). Our analysis of transcriptomic data revealed that genes and enzymes associated with these three pathways produced a transcriptional regulatory response in chrysanthemum; therefore, these components may also be the major regulators of HSR in chrysanthemum. However, the precise responses of these pathways to high temperature and mechanisms underlying the regulation of flower color in chrysanthemum remain unclear.

### What is the link between anthocyanin synthesis and high temperature stress?

Anthocyanin biosynthesis is regulated by an array of enzymes. Genes encoding these enzymes, namely *CHS, CHI, DFR, F3H, ANS, 3GT*, and *F3'H*, are called structural genes related to anthocyanin metabolism (Willits et al., 2005; Zhang et al., 2014). Our transcriptome analysis revealed that the expression of anthocyanin synthesis-related genes was significantly downregulated (Figures 6A, 7), consistent with qRT-PCR results, and the total anthocyanin content also decreased under high-temperature stress. In addition, *CmHY5* (Unigene19768_All), a bZIP transcription factor, was significantly downregulated. HY5 is known to regulate flower color in apples, and its association with BBXs, COP1, and MYBs has been demonstrated in rice (Luo et al., 2018), *Arabidopsis* (Gangappa et al., 2013), and apple (Liu et al., 2019). Here, we used qRT-PCR to verify HY5 downregulation and to further investigate the molecular mechanism underlying the regulation of flower color under high-temperature stress. In plants, structural genes involved in anthocyanin biosynthesis are primarily regulated by a class of conserved MBW complexes (Jin et al., 2016; Goswami et al., 2018). Among these, the MYB and bHLH transcription factors play major regulatory roles. Specifically, the MBW protein complex directly regulates anthocyanin content in plants by activating or inhibiting the transcription of structural genes related to anthocyanin synthesis (Nabavi et al., 2020). In our analysis, eight MYB transcription factors were downregulated (Figure 6B). These results are consistent with reports that high-temperature stress inhibited the expression of *MdMYB4, MdMYB16, MdMYB17*, and *MdMYB114* and affected the synthesis of anthocyanin in apples (Lin-Wang et al., 2011). *MpMYBS3* is highly expressed in response to low-temperature signals (Dou et al., 2016), and our analysis showed that *CmMYBS3* (CL9278.Contig2_All) was downregulated under high-temperature stress; hence, CmMYBS3 may be involved in high-temperature stress response. *PpMYB114* promotes the expression of *PpUFGT* involved in anthocyanin metabolism (Ni et al., 2019). In the present study, *CmMYB114* (Unigene1147_All) and *Cm3GT* (CL8501.Contig3_All) were downregulated after high-temperature treatment, indicating that a similar regulatory network may exist in chrysanthemum. In *Petunia hybrida, PhPAP1* positively regulated the anthocyanin structural gene *PhCHS*, and the overexpression of *PhPAP1* and *PhCHS* led to abundant anthocyanin accumulation after 12 h of UV-A irradiation (Matoušek et al., 2006). Moreover, in the ethylene-insensitive *Arabidopsis* mutant *ein2-1*, abundant sucrose-induced anthocyanins were accumulated at high temperature through elevated PAP1 transcription (Kwon et al., 2011). These results are consistent with *CmPAP1* (Unigene28293_All) downregulation after high-temperature treatment in our experiment. In chrysanthemum, a large amount of ethylene may be synthesized under high-temperature stress, which downregulates downstream *CmPAP1* expression and hinders anthocyanin accumulation. Furthermore, PAP1 and PAP2 can bind the COP1/SPA complex and positively control anthocyanin accumulation in *Arabidopsis* (Maier et al., 2013). Our data showed that PAP2 (Unigene42013_Al), COP1 (Unigene24944_All), and SPA4 (CL5840.Contig2_All) were downregulated, indicating a similar regulatory mechanism in chrysanthemum. ANT1 can form a complex with EGL3, GL3, and TTG1 to positively regulate anthocyanin metabolism (Petroni and Tonelli, 2011), which is consistent observation that ANT1 (Unigene52085_All) was downregulated in chrysanthemum at high temperature.

Furthermore, bHLH transcription factors affect flower color. In particular, PhAN1 can interact with MYB and WD to positively regulate anthocyanin synthesis and activate various signaling pathways through interaction with MYB transcription factors (Quattrocchio et al., 2006; Petroni and Tonelli, 2011). In *Arabidopsis thaliana*, the WD protein TTG1 acts in concert with the GL3, EGL3, or TT8 proteins as well as the R2R3–MYB transcription factors PAP1, PAP2, MYB113, and MYB114 (Zhang et al., 2003; Zimmermann et al., 2004; Gonzalez et al., 2008). In *Freesia hybrida* Klatt, FhTTG1 encodes the WD40 protein, which forms the MBW complex and regulates anthocyanin or procyanidin biosynthesis, the specificity of which depends, in part, on the interacting proteins (Yamagishi et al., 2010; Verweij et al., 2016; Qi et al., 2022). Our data showed that the bHLH transcription factor AN1 (Unigene52085_All) and the WD40 protein TTG1 (CL5816.Contig5_All) were downregulated; these proteins may interact with MYB transcription factors to form the MBW complex and reduce anthocyanin synthesis under high-temperature stress. In addition, MADS-box transcription factors have been found to be involved in anthocyanin biosynthesis in a variety of plants, such as apple (Onik et al., 2018), strawberry (Lu et al., 2018), and sweet cherry (Qi et al., 2020). Recently, studies have shown that ScAG and ScAGL11 as negative regulators of anthocyanin biosynthesis in *Senecio cruentus* ray florets, which influence bicolor pattern formation (Qi et al., 2022). In our results, *CMB1-like* (Unigene34995_All), *AGL15* (Unigene19595_All), *AGL3* (Unigene40538_All), and *SOC1* (Unigene31972_All) were significantly downregulated after high temperature stress. It is speculated that MADS-box transcription factors are involved in anthocyanin biosynthesis in chrysanthemum, but the specific molecular mechanism needs to be further studied.

In summary, chrysanthemum response to high temperature may involve the following three pathways: (1) extracellular Ca^2+^ influx promotes Ca^2+^ channels and signaling responses, which in turn promotes the expression of downstream MAPKs and transmits the high-temperature signal to the nucleus; (2) ROS production and accumulation of leads to a series of complex heat stress response pathways, such as HSF and HSP synthesis and NO accumulation, which in turn activates the plant's own immune stress response; and (3) structural genes involved in anthocyanin synthesis are downregulated, thereby inhibiting anthocyanin production and leading to flower discoloration under high temperature in chrysanthemum. These structural genes are regulated by different MBW protein complexes and other transcription factors that affect anthocyanin biosynthetic pathways in plants. However, although we assessed the expression of genes related to plant response to high temperature and anthocyanin metabolism through transcriptome analysis, response to heat stress and regulation of anthocyanin metabolism in plants involve complex regulatory networks. The links among these genes and their respective molecular functions remain clear and warrant further exploration.

## Conclusion

**Figure 8:**
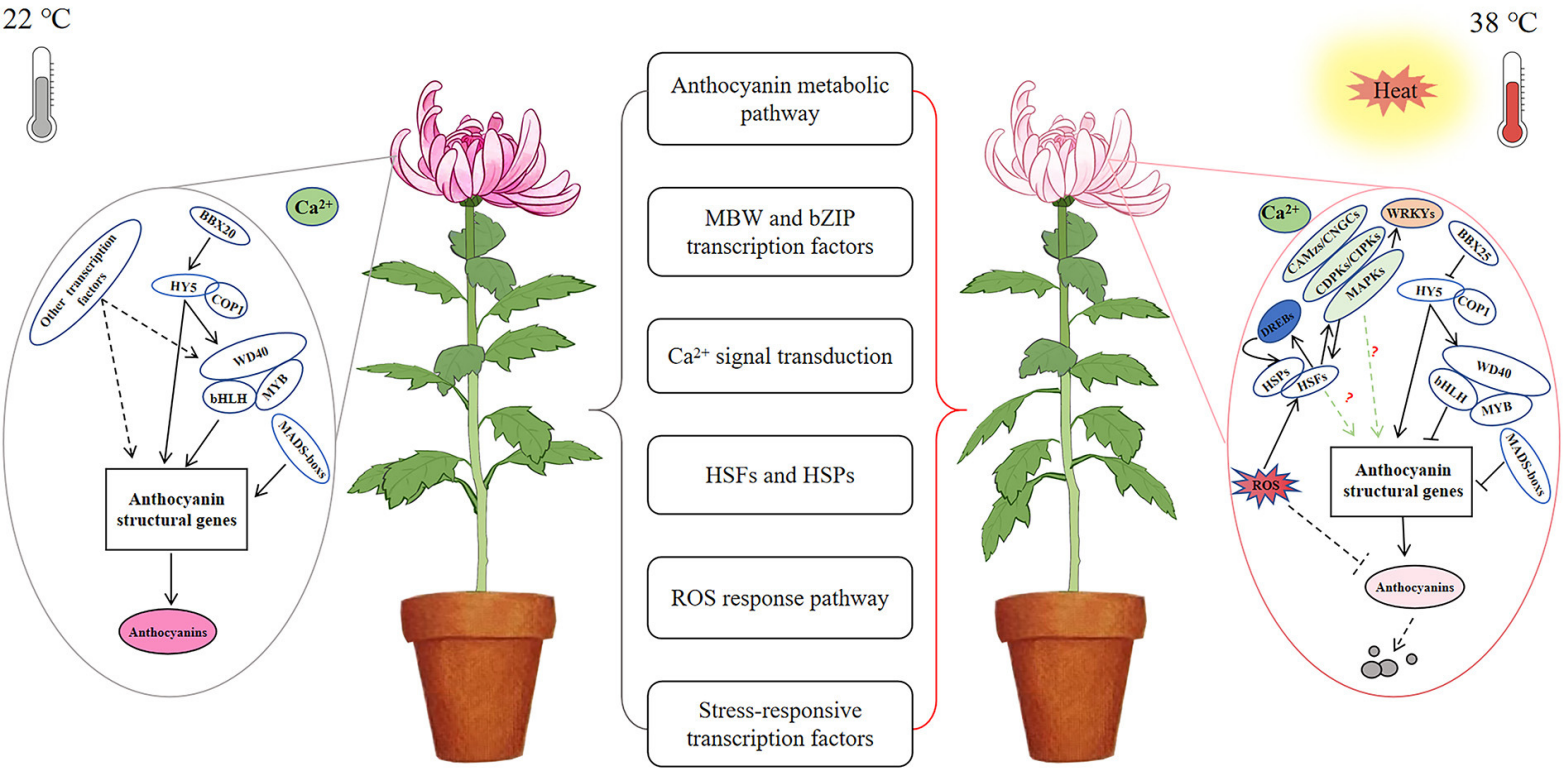
A possible regulatory network of chrysanthemum flower color under high temperature stress. Anthocyanin biosynthesis was normal at 22°C; anthocyanin biosynthesis was inhibited and accelerated degradation under high temperature stress at 38°C.

The discoloration phenotype of chrysanthemums under high-temperature stress is directly related to the inhibition of anthocyanin synthesis. However, multiple pathways may be involved in response to high-temperature stress, indicating the presence of a complex molecular regulatory network involving anthocyanin metabolism in chrysanthemums (Figure 8). Overall, our preliminary transcriptomic analysis revealed the regulation of flower color, as affected by high-temperature stress, although the precise molecular mechanism should be further studied.

## Data availability statement

The datasets generated for this study can be found in the NCBI sequence reads archive (SRA) database under BioProject No. PRJNA859415.

## Author contributions

JJ designed the experiments. XH and JQ performed the experiments. ZS and XH analyzed the data. ZS, XH, and GW wrote the manuscript. L-jZ, SC, WF, and FC discussed the results and commented on the manuscript. L-jZ and ZS provided the funds. All authors contributed to the article and approved the submitted version.

## Funding

This work was financially supported by the Hainan Provincial Natural Science Foundation of China (322QN340), the special research fund for doctoral students of Sanya Yazhou Bay Science and Technology City (HSPHDSRF-2022-07-002), and the Guiding Fund Key Projects for Sanya Institute of Nanjing Agricultural University (NAUSY-ZD03).

## Conflict of interest

The authors declare that the research was conducted in the absence of any commercial or financial relationships that could be construed as a potential conflict of interest.

## Publisher's note

All claims expressed in this article are solely those of the authors and do not necessarily represent those of their affiliated organizations, or those of the publisher, the editors and the reviewers. Any product that may be evaluated in this article, or claim that may be made by its manufacturer, is not guaranteed or endorsed by the publisher.
